# Electrical conductivity in a non-covalent two-dimensional porous organic material with high crystallinity†

**DOI:** 10.1039/d0sc05602b

**Published:** 2021-01-14

**Authors:** Qizhi Xu, Boyuan Zhang, Yihang Zeng, Amirali Zangiabadi, Hongwei Ni, Rongsheng Chen, Fay Ng, Michael L. Steigerwald, Colin Nuckolls

**Affiliations:** The State Key Laboratory of Refractories and Metallurgy, Institute of Advanced Materials and Nanotechnology, Wuhan University of Science and Technology Wuhan 430081 China cn37@columbia.edu chenrs@wust.edu.cn; Department of Chemistry, Columbia University New York New York 10027 USA; Department of Physics, Columbia University New York New York 10025 USA; Department of Applied Physics and Applied Mathematics, Columbia University New York New York 10027 USA

## Abstract

Electroactive macrocycle building blocks are a promising route to new types of functional two-dimensional porous organic frameworks. Our strategy uses conjugated macrocycles that organize into two dimensional porous sheets *via* non-covalent van der Waals interactions, to make ultrathin films that are just one molecule thick. In bulk, these two-dimensional (2D) sheets stack into a three-dimensional van der Waals crystal, where relatively weak alkyl–alkyl interactions constitute the interface between these sheets. With the liquid-phase exfoliation, we are able to obtain films as thin as two molecular layers. Further using a combination of liquid-phase and mechanical exfoliation, we are able to create non-covalent sheets over a large area (>100 μm^2^). The ultrathin porous films maintain the single crystal packing from the macrocyclic structure and are electrically conductive. We demonstrate that this new type of 2D non-covalent porous organic framework can be used as the active layer in a field effect transistor device with graphene source and drain contacts along with hexagonal boron nitride as the gate dielectric interface.

## Abstract 2

Ultrathin porous films held together by non-covalent van der Waals interactions was obtained by a top-down approach, which is then utilized as channel material in a two-dimensional planar field-effect transistor device through easy stamp transfer.

## Graphical abstract

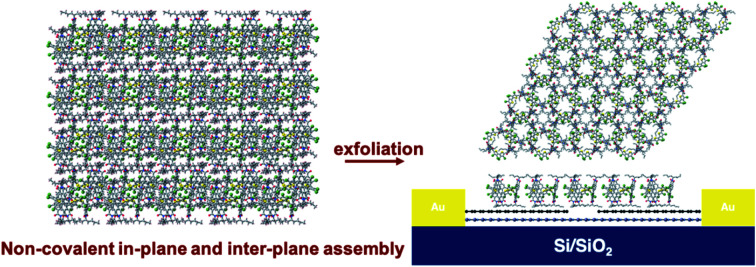

We describe a new type of two-dimensional (2D), molecularly-thin porous organic framework that is formed from macrocyclic building blocks that assemble, through non-covalent interactions, into a porous two-dimensional plane. Covalent organic frameworks (COFs) are promising in applications due to their ability to host other functional molecules in the voids.^1–7^ Many porous frameworks have been demonstrated to be useful in energy storage,^8^ catalysis,^9–11^ separation,^12,13^ optoelectronics^4,14^ and sensing.^15,16^ In order to construct nanodevices with porous channels, ultrathin films of porous frameworks has been prepared with bottom-up^4,17–20^ and top-down^1,21^ approaches. The top-down approaches to these materials are enabled by strong covalent bonds in the two-dimensional plane and weak van der Waals interactions between them, similar to what is seen in two-dimensional materials such as graphene and TMDs.^22–27^ For porous ultrathin films, the electrical conductance has not been extensively investigated.^2,7,20,28,29^

Here, we explore making molecularly thin layers in which conjugated macrocycles are used as building blocks and non-covalent van der Waals interactions are the adhesive that assembles these molecules into rigid, porous layers. By adjusting the relative strengths of the interactions that direct the assembly within the plane and those holding the two-dimensional layers with respect to each other, we can exfoliate these non-covalent porous frameworks using the same means employed for traditional two-dimensional van der Waals materials.^30^ Using liquid-phase and mechanical exfoliation, we create porous films that are as thin as two-layers of molecules. These new results are exciting and useful because previously we were not able to obtain such high-ordered thin porous film directly from its bulk crystal and were limited to investigating the electronic properties of this hollow organic capsules in spin-coated films. These ultrathin porous films are ordered over large areas and maintain the single crystal packing from the macrocyclic building blocks. To demonstrate the utility of this new type of ultrathin material, we fabricated 2D field effect transistor (FET) devices in which graphene is the source/drain contacts, hexagonal boron nitride is the gate dielectric interface, and the exfoliated molecular sheet is the active layer. These ultrathin self-assembled materials are efficacious at transporting electrons and will find utility in gas sensing and applications similar to traditional two-dimensional materials.

Fig. 1 displays the molecular building block (**1**). Characterization is contained in the ESI† and a previous report.^31^**1** has several important molecular features in its solid-state assembly. It is a rigid and shape persistent macrocycle that has an interior and an exterior (Fig. 1a), and in bulk, has a pore of ∼11.4 Å in diameter and a surface area of 20 m^2^ g^−1^ from BET measurements.^31^ When it assembles in the crystalline state, it forms two-dimensional porous sheets with two types of cavities (Fig. 1b), one molecule thick, that are held together by relatively strong π–π contacts and Br–PDI interaction between the bromine atoms on the thiophenes and adjacent PDI molecules (Fig. 1c), which plays a crucial role in the self-assembly of the films. The close proximity of the molecules in the 2D plane together with the conjugation within the macrocycle facilitate charge transport of electrons in the 2D plane. These electrically conductive porous sheets then stack into a three-dimensional crystal in which adjacent sheets are separated from one another by the alkane sidechains of the perylene diimide (Fig. 1d). It is in this alkane gallery that we see an opportunity for exfoliation to yield ultrathin 2D sheets.

**Fig. 1:**
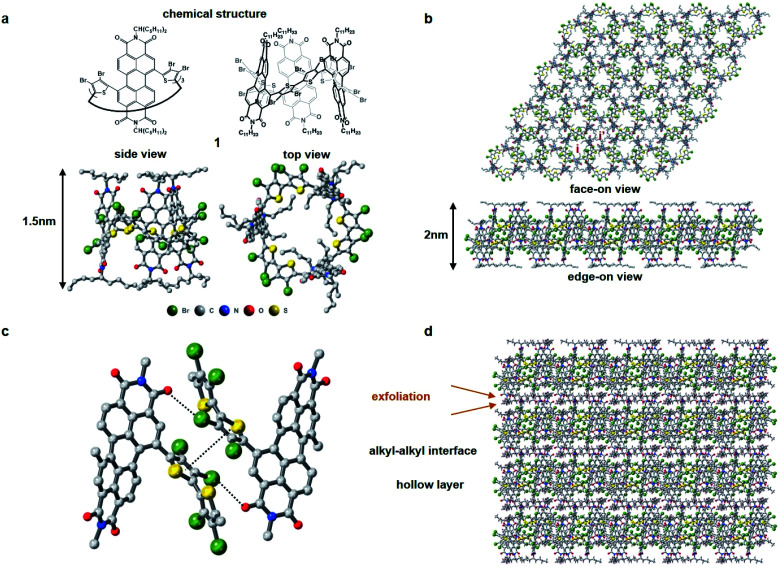
Structure and packing of molecule **1**. (a) Chemical structure, side view and top view of molecular structure of **1**. C, N, O, S, Br are colored in grey, blue, red, yellow and green, respectively. The vertical distance of one macrocycle is about 1.5 nm. (b) Face-on view and edge-on view of one layer of **1**. The internal cavity of **1** and the cavity formed by the packing of **1** are labeled as i and i′, respectively. (c) Interactions binding two adjacent macrocycles from neighboring brominated thiophene rings. (d) View of packing of **1** along the *c* axis through the interaction of alkane sidechains. Exfoliation takes place at the alkyl–alkyl interface. One layer of **1** is about 2 nm in thickness.

We isolated crystals of this material that were grown from solution and then tested whether they can be exfoliated. Fig. 2a displays a representative micrograph of one of the crystals. The crystal has a pseudo-hexagonal packing of the molecular building blocks in the two-dimensional plane, and this symmetry is mirrored in the hexagonally-shaped crystals. The simplest method for exfoliation is mechanical exfoliation, which is most commonly performed using scotch-tape.^32,33^ We place the single crystals onto clean scotch-tape and repeat the mechanical exfoliation process for a few repetitions, and then we transfer the exfoliated crystals onto a clean silicon wafer. Fig. 2b displays an atomic force microscopy (AFM) micrograph of the typical non-covalent porous 2D sheet of **1** we obtained from this method. The porous sheets are flat and smooth and a few micrometers in diameter with a thickness of ∼8 nm; this thickness corresponds to a stack of five molecular layers of **1**. This result demonstrates that non-covalent interactions are strong enough to hold molecules together to form ultrathin porous materials. Just as with traditional two-dimensional materials, the non-covalent porous organic 2D sheets of **1** are flexible as evidenced by the wrinkles and folds in the micrograph in Fig. 2b and S2.†

**Fig. 2:**
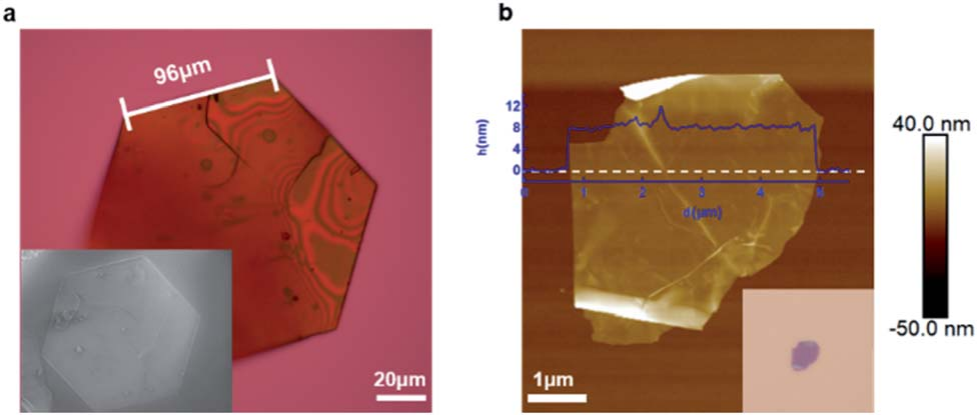
Mechanical exfoliation of **1**. (a) Optical microscopy and scanning electron microscope (SEM) (inset) images of a single crystal of macrocycle **1**. (b) AFM and optical microscope (inset) images of the ultrathin non-covalent porous sheet of **1** on a silicon wafer obtained from mechanical exfoliation.

We were unable to obtain porous films as thin as a single layer and also large-area samples using mechanical exfoliation, and thus we next explored if liquid-phase exfoliation^34,35^ could produce them. Because the halogen bonds that hold the sheets together should be most robust in solvents with a low-dielectric constant that lack heteroatoms, and because the groups holding the sheets together are the alkane sidechains, we chose saturated hydrocarbons (hexane or heptane) as the solvents for exfoliation. Fig. 3a shows the process we follow for the liquid-phase exfoliation. We suspend single crystals of **1** in heptane and sonicate the mixture for five minutes. We drop cast the supernatant solution on silicon wafer and examine them with AFM. Remarkably, we are able to obtain non-covalent porous organic frameworks as thin as only two molecular layers (Fig. 3b).^36^ Nevertheless, the lateral size of the porous 2D sheets of **1** we could obtain using this method are quite small, making it difficult to fabricate devices from them.

**Fig. 3:**
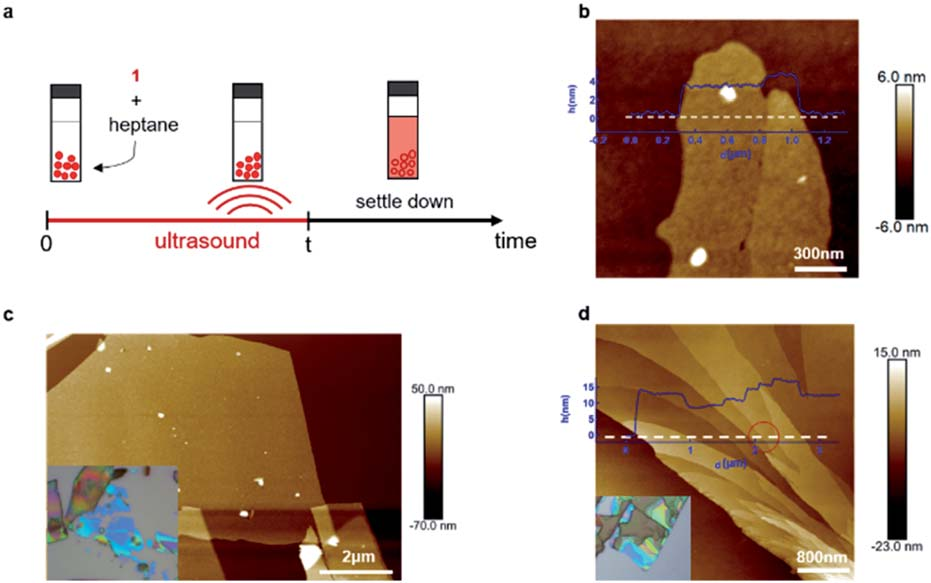
Liquid-phase exfoliation and combination of liquid-phase and mechanical exfoliation. (a) Schematic showing the liquid-phase exfoliation process. (b) AFM image of the ultrathin sheet of **1** on a silicon wafer obtained from liquid-phase exfoliation method. The sheets in this micrograph are two molecular layers (left) and three molecular layers (right) in thickness. (c) AFM image of the ultrathin sheet of **1** on a silicon wafer obtained from combination of liquid-phase and mechanical exfoliation, inset: optical microscope image of the ultrathin sheet **1**. (d) AFM image showing the height change across the ultrathin sheet of **1** on silicon wafer obtained with combination methods, inset: optical microscope image of the ultrathin sheet of **1**.

To get large-area films characteristic of the mechanical exfoliation and thin films characteristic of the liquid-phase exfoliation, we combined the two methods. We first immerse the crystal of **1** in heptane for a few minutes to let the solvent seep into the gallery between the sheets and weaken the interlayer interactions. Then, we use mechanical exfoliation to isolate the ultrathin films. With this method, we obtained sheets of **1** with a lateral size of over ten micrometers, as shown in Fig. 3c. By carefully examining the exfoliated non-covalent porous 2D sheets of **1**, we were also able to observe the height change across the sheet (Fig. 3d) with integer values of the layer thickness after the exfoliation steps. As marked red in Fig. 3d, we could identify a single layer of **1** with a height difference between these two surfaces of about 1.5 nm, corresponding to monolayer of molecule **1**.

We conducted transmission electron microscopy (TEM) studies to characterize the crystallinity of the as-prepared non-covalent porous 2D sheets of **1**. As shown in Fig. 4a (inset), the 2D sheets exhibit a layered structure after liquid-phase exfoliation. The selected area electron diffraction (SAED) in the area (marked by the red circle) reveals a hexagonal diffraction pattern, with the bright reflections corresponding to the (2 1(−) 0) plane, with a spacing of 11.3 Å. This diffraction pattern confirms that the non-covalent, porous 2D sheets of **1** retain the single crystal packing and are stable to the liquid-phase processing.

**Fig. 4:**
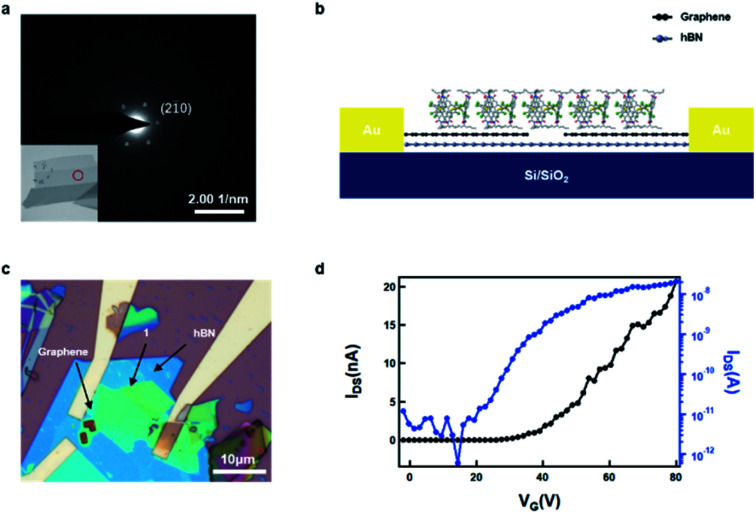
TEM characterization and device fabrication. (a) SEAD pattern and TEM image (inset) of the non-covalent porous ultrathin sheet **1** obtained by liquid-phase exfoliation. (b) Schematic showing the structure of the hBN/Graphene/**1** device based on the non-covalent porous ultrathin sheet **1** with graphene as electrodes and hBN as the dielectric layer. (c) Optical microscope image showing the as-fabricated hBN/Graphene/**1** device. (d) Transfer curve of the hBN/Graphene/**1** device.

We next sought to determine the ability of these non-covalent porous ultrathin layers to transport charge. Because these films are van der Waals materials, we sought to make devices with van der Waals interfaces. [The ESI† contains the current/voltage curves for **1** in a more traditional organic FET with Au contacts and trichloro(octadecyl)silane coated SiO_2_ as the gate dielectric.] The source-drain contacts were fabricated from graphene and the dielectric interface was hexagonal boron nitride (hBN). A schematic of the device is shown in Fig. 4b. To create this device, we first exfoliate graphene and hBN onto a silicon substrate. We then follow a published procedure^37^ to first pick up hBN and then graphene to make the hBN/graphene stack. We transfer this hBN/graphene stack onto another clean silicon substrate. Then the graphene was cut using electron beam lithography and an oxygen plasma to open a 300 nm gap between graphene electrodes (see Fig. S4† for the AFM details of graphene electrodes). In order to transfer the non-covalent porous ultrathin sheets of **1** onto the graphene electrodes, we exploit the combined liquid/mechanical exfoliation method above to obtained 2D sheet of **1** with polydimethylsiloxane (PDMS) polymer as the substrate, which was then used for the stamp transferring. In this manner, we were able to transfer the ultrathin sheets (∼20 nm) onto the graphene electrodes. Fig. 4c displays the optical microscope image of the device, and Fig. 4d displays the FET transfer curves revealing that the material is an efficacious, n-type transistor. Several features of the device are noteworthy. The electron mobility in the linear regime was estimated to be 1.6 × 10^−4^ cm^2^ V^−1^ s^−1^ from the transfer curve. As expected, it is somewhat lower than the electron mobility estimated from the saturation regime of the traditional OFET shown in Fig. S3.†^38^ Despite the small size and the nanoscale thickness, the material exhibits over 3 orders of magnitude difference in current between the off and on state of the device. The threshold voltage is about 39 V, implying that the device turns on at relatively high voltage. We surmise that the contact, through the alkane sidechains is an impediment to more efficient charge transport.

## Conclusions

We have described here a new type of porous 2D organic framework held together by non-covalent bonding and charts a clear path to creating functional non-covalent porous 2D materials with tunable properties by increasing intermolecular forces within the plane relative to those between the planes. Through a combination of liquid-phase and mechanical exfoliation of the molecular crystal we are able to make non-covalent porous 2D sheets that can be as thin as two-layer molecules over a large area. These sheets are able to transport electrons in a field effect transistor device with graphene source-drain contacts and hBN gate dielectric interfaces. Because these materials are electrically conductive and retain the single crystal structure from macrocyclic building blocks, they will find utility as reaction chambers for studying chemistry in confined, and electrified spaces, as molecular sensors, and as electrically activated sieves.

## Conflicts of interest

There are no conflicts to declare.

## Supplementary Material

SC-012-D0SC05602B-s001
