# Supplementary material for: Electrical conductivity in a non-covalent two-dimensional porous organic material with high crystallinity
Source: Chem Sci. 2021 Jan 14;12(8):2955–9. doi: 10.1039/d0sc05602b (PMC8179372; doi:10.1039/d0sc05602b)
Supplement: SC-012-D0SC05602B-s001 [file SC-012-D0SC05602B-s001.pdf]

Electronic Supplementary Material (ESI) for Chemical Science.
This journal is © The Royal Society of Chemistry 2021

Supporting information for:

Electrical Conductivity in a Non-Covalent Two-dimensional Porous Organic Material with High Crystallinity

Qizhi Xu[1,2,5], Boyuan Zhang[2,5], Yihang Zeng[3], Amirali Zangiabadi[4], Hongwei Ni[1], Rongsheng Chen[1,*], Fay Ng[2], Michael L. Steigerwald[2], Colin Nuckolls[2,*]

[1]The State Key Laboratory of Refractories and Metallurgy, Institute of Advanced Materials and Nanotechnology, Wuhan University of Science and Technology, Wuhan 430081, China

[2]Department of Chemistry, Columbia University, New York, New York 10027, United States

[3]Department of Physics, Columbia University, New York, New York 10025, USA

[4]Department of Applied Physics and Applied Mathematics, Columbia University, New York, New York 10027, United States

[5]These authors contributed equally

*Email: cn37@columbia.edu, chenrs@wust.edu.cn

Table of Contents

1. **General information** **2**
2. **Characterization of Macrocycle 1** **3**
3. **AFM image of 2D sheet of 1** **4**

4. **Fabrication of traditional organic field-effect transistor** **4**

5. **Fabrication of coplanar field effect transistor** **5**

1. General information

Materials

Commercial reagents were used without further purification.

Synthesis

Macrocycle **1** is synthesized according to reference: Nuckolls, C. *et al.* Hollow organic capsules assemble into cellular semiconductors. *Nat. Commun.* **9**, 1957 (2018). The crystals were grown using vapor diffusion: in a small vial, a concentrated solution of macrocycle **1** (15 mg macrocycle 1 in 1 mL chlorobenzene) was placed in a jar with ethanol. The jar was sealed and left at ambient temperature for 10 days.

Instrumentation

Scanning electron microscopy was performed with Zeiss Sigma VP Scanning Electron Microscope at 1.0 kV. Optical microscope images were obtained with Nikon Eclipse LV150N. Atomic force microscopy measurements were carried out in scan-analyst mode on a Bruker Multi-Mode AFM at ambient conditions. Transmission electron microscopy and selected area electron diffraction was performed with FEI Talos F200X. E-beam lithography was performed with Nanobeam nB4 and evaporation was performed with Angstrom evaporator. The device characteristics were measured in the glovebox with nitrogen atmosphere at room temperature with an Agilent 4155C

semiconductor parameter analyzer.

2. Characterization of Macrocycle 1

Thermogravimetric Analysis (TGA)

TGA curve (Figure S1) of macrocycle **1** was recorded using a TA Q500 thermal analysis system under nitrogen flow, which shown in Figure S1. From the TGA result, we found that the hydrocarbon side chains and bromine atoms of the macrocycle **1** were removed around 350°C.

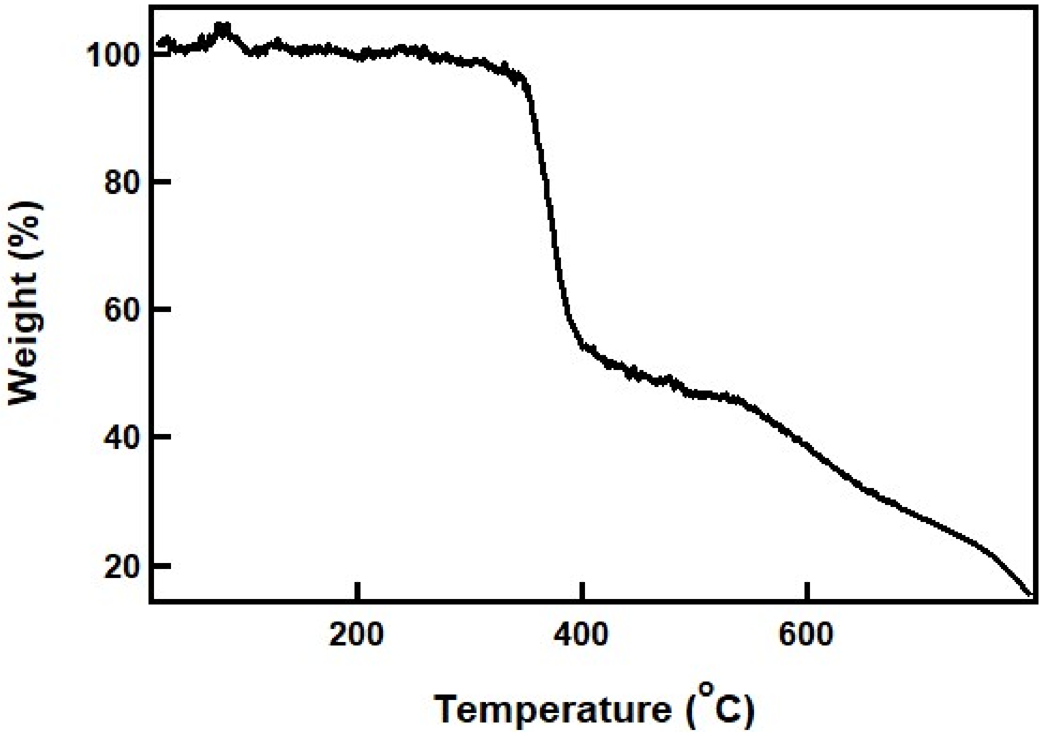

Figure S1: TGA curve of macrocycle **1.**

3. AFM image of non-covalent porous 2D sheet of 1

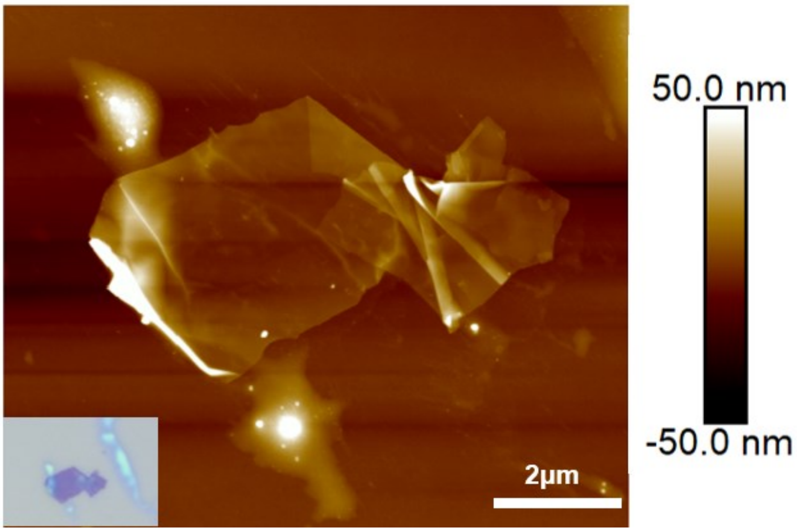

Figure S2: AFM and optical microscope (inset) image of the non-covalent porous 2D sheet of **1** obtained from the mechanical exfoliation on a silicon wafer.

4. Fabrication of traditional organic field-effect transistor (OFET)

To fabricate the traditional OFET device, we used degenerately doped silicon wafer with a 285 nm thermal silicon oxide dielectric layer as a common back gate. We first modify it with octadecyltrichlorosilane (OTS) according to previous reported procedures. We then use the combination exfoliation method to yield ultrathin porous sheets of **1** directly on the OTS modified substrate. Next, we use a stencil mask to deposit top-contact, source and drain electrodes (Ti/Au, 3 nm/50 nm) with a channel length of 20 μm. The samples were annealed under inert atmosphere at 160℃ for 10 minutes to optimize device performance. Figure S3 show the typical transfer curve of a device fabricated with non-covalent porous 2D sheets of 1 exhibiting n-type transport behavior and an electron mobility of 6.21 x 10^{-4} $cm^2V^{-1}s^{-1}$.

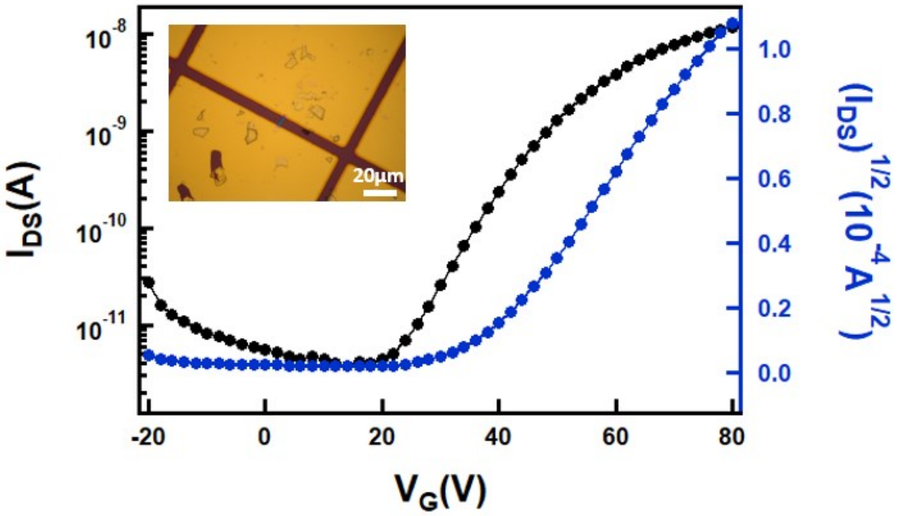

Figure S3: Transfer curve and optical microscope image (inset) of a traditional OFET device with the non-covalent porous 2D sheet of **1** as the active layer.

5. Fabrication of coplanar field effect transistor

We first use the previous reported method to transfer the hBN/graphene stack on a clean silicon wafer. Briefly speaking, we first make a polydimethylsiloxane(PDMS)/ polypropylene carbonate(PPC) stamp, then use it to pick up the exfoliated hBN on the silicon wafer. Following that, we pick up the desired exfoliated graphene with the PDMS/PPC/hBN stack. Then the whole PPC/hBN/Graphene stack was transferred onto a clean silicon wafer and PPC was removed by vacuum annealing at 350°C for 20min. E-beam lithography (PMMA A4 495k + A6 950k), evaporation and oxygen plasma were then used to fabricate the graphene electrodes with an opening of 300nm in the center (Figure S4). Finally, we transferred the exfoliated non-covalent porous 2D sheet of **1** on the PDMS onto the graphene electrodes. The output curve of the hBN/graphene/**1** transistor is shown in Figure S5.

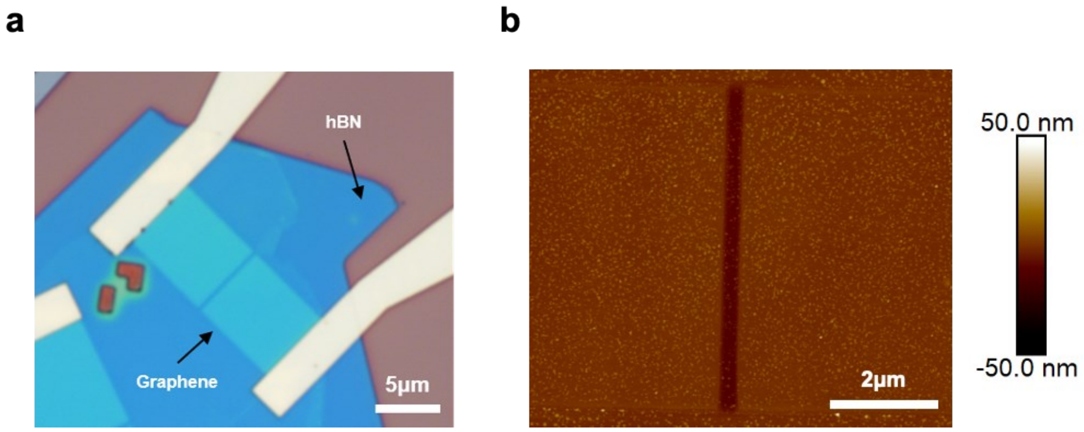

Figure S4: a) Optical microscope image of hBN/Graphene stack with an opening gap of 300nm in the center on a silicon wafer. b) AFM image of hBN/graphene stack showing the 300nm gap in the center.

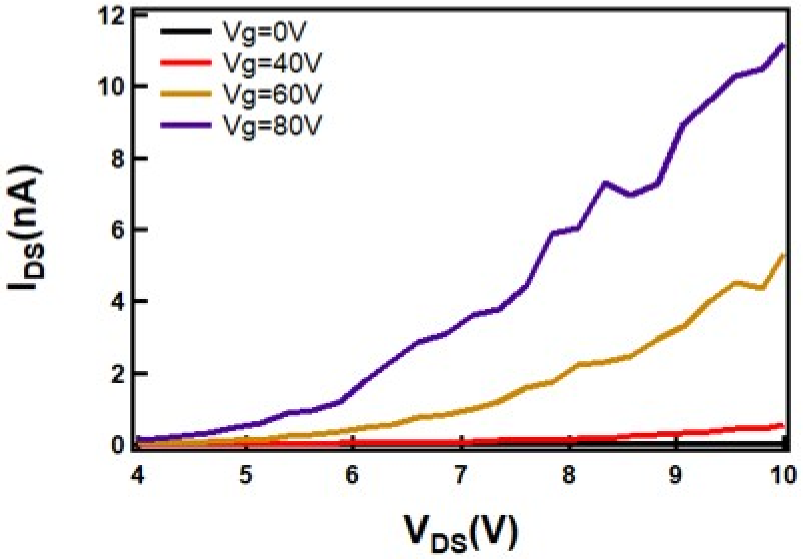

Figure S5: Output curve of hBN/Graphene/**1** device on the silicon wafer.
